# Changes in Circadian Variations in Blood Pressure, Pain Pressure Threshold and the Elasticity of Tissue after a Whole-Body Photobiomodulation Treatment in Patients with Fibromyalgia: A Tripled-Blinded Randomized Clinical Trial

**DOI:** 10.3390/biomedicines10112678

**Published:** 2022-10-23

**Authors:** Santiago Navarro-Ledesma, James Carroll, Ana González-Muñoz, Leo Pruimboom, Patricia Burton

**Affiliations:** 1 Faculty of Health Sciences, Campus of Melilla, Department of Physiotherapy, University of Granada, 52004 Melilla, Spain; 2 THOR Photomedicine Ltd., Chesham HP5 1LF, UK; 3 Clinica Ana Gonzalez, 29018 Malaga, Spain; 4 PNI Europe, 2518 JP The Hague, The Netherlands

**Keywords:** fibromyalgia, pain, chronic pain, chronobiologic indicators, circadian rhythm disorders, blood pressure, autonomic symptoms, musculoskeletal disorders, elastography

## Abstract

This study analysed circadian variation changes in blood pressure (BP), the pain pressure threshold (PPT) and the elasticity of tissue in patients with fibromyalgia (FM) after a whole-body photobiomodulation (PBM) treatment. This was a tripled-blinded randomized clinical trial including forty participants with FM. Participants using validated self-measurement BP devices attained readings that were used to calculate the circadian variation. Additionally, a standard pressure algometer of 1cm2 was used to assess 13 tender points by exerting a pressure of up to 4 kg, and strain elastography assessed the elasticity of tissue. Circadian variations in BP showed significant differences after the PBM intervention (*p* = 0.036). When comparing PPT between groups, statistically significant differences were found in the occiput (*p* = 0.039), low cervical (*p* = 0.035), trapezius (*p* = 0.037), second rib (*p* < 0.001) and medial epicondyle points (*p* = 0.006). Furthermore, there were statistically significant differences in both the trapezius and the forearm at the distal dorsal third SEL values (*p* ≤ 0.001) when comparing groups. Whole-body PBM produces changes in circadian blood pressure, the pain pressure threshold and the elasticity of tissue after a treatment program was carried out. However, more studies are needed to corroborate our findings as well as to better understand the underlying mechanisms.

## 1. Introduction

Chronic musculoskeletal pain (CMP) is one of the most common forms of chronic pain and has a profound impact on individuals and on society [1]. Fibromyalgia (FM), considered to be the most frequent cause of diffuse chronic musculoskeletal pain, is a multicomponent illness with an unknown etiology [2]. The American College of Rheumatology (ACR) included criteria, such as pain sensitivity to 4 kg of digital pressure and pain of a diffuse nature (both lacking explanation by degenerative or inflammatory disorders), cognitive behavioral disorders, fitful sleep, tiredness and somatic symptoms in the diagnosis of FM [3,4]. Although it is more common in middle-aged adults, any age group can be affected [5], with a range from 0.5% to 5% in the general population and up to 15.7% in a clinical setting. Despite its worldwide prevalence, a complete understanding of its etiology and pathogenesis remains unclear.

A multifactorial understanding of FM is crucial in the development of new alternative treatments. An altered central nervous system response has been shown to exist in those suffering from FM, which may explain the association of FM with cardiovascular risks and explain a state of low-grade inflammation [6] and its interplay with altered regions of the brain which process pain and behaviour [1,7,8,9]. In this regard, patients suffering from FM have been shown to present an alteration in circadian blood pressure (BP) and persistent nocturnal sympathetic hyperactivity, which leads to the autonomic nervous system malfunctioning. Furthermore, patients with FM have been shown to exhibit a nondipping BP pattern [10]. In healthy subjects, a higher BP is known to be associated with hypoalgesia; however, this has not been found in FM [11]. Furthermore, descending inhibitory pain mechanism deficiencies in these patients would confirm the absence of hypoalgesia in patients with chronic pain [12].

A disrupted circadian clock has been associated with decreased health conditions [13] and can be present in metabolic syndromes [14]; neurodegenerative diseases [15,16]; and inflammatory diseases [17,18], including some types of cancer [17,19]. Furthermore, the presence of both central and peripheral sensitization has been shown to cause vegetative alterations such as myofascial pain, abnormalities in the diameter and density of capillaries, changes in the sympathetic tone and activity of the primary muscular blood vessels, mechanotransduction, microcirculatory blood flow, skin conductance and the tensegrity of the tissues [20,21]. In this context, ultrasound elastography (SEL) is used to quantify the elasticity of tissue, such as in the tender points by assessing both stiffness and tissue quality [22].

Therefore, alterations in circadian BP appear to be an additional risk factor in patients with FM, and treatments which focus on recovering BP patterns may be indicated, along with the assessment of tissue elasticity and pain pressure threshold (PPT).

Circadian rhythms throughout the body are synchronized by the “master clock”, namely, the suprachiasmatic nucleus (SCN), via the retinohypothalamic path; hence, the diurnal/nocturnal BP ratio allows variations in blood pressure (BP) to be used as a method of assessing this clock [23]. Neurohumoral factors that affect cardiovascular systems and the autonomic nervous system are affected when an alteration of the circadian rhythm appears, which present chronic changes in the BP pattern [24,25], and could additionally contribute to the pathogenesis of chronic disorders. Furthermore, the alteration of circadian rhythms confirms the presence of mitochondrial dysfunction facilitating changes in the gut microbiome, immune function, and autonomic nervous system, finally worsening mental health. In this regard, a relationship between psychological factors and vegetative symptoms has been shown in patients with FM [26]. Shedding light on potential treatments which improve circadian rhythms and vegetative symptoms can help in better understanding and treating those suffering with FM.

Photobiomodulation (PBM) therapy, previously named low-level laser therapy (LLLT), is a developing, non-invasive therapy for those suffering from FM. It has been proven to be effective in improving musculoskeletal and neuropathic pain in these patients, resulting in an improvement in the quality of their life [27]. At wave lengths shorter than 600 nm, hemoglobin and melatonin, the main chromophores in tissue, demonstrate high absorption bands. Hence, the currently established effective wave length for PBM, where tissue penetration is maximised, ranges from 600 to 1070 nm, with the fluence (energy density) ranging between 1 and 20 J/cm^2^. The wave length range of 600–700 nm is used for superficial tissue treatments, and wave lengths ranging from 780 to 950 nm, which penetrate further because they are longer, can treat deeper-seated tissues [28,29,30].

The biostimulation target for PBM could be the mitochondria [31], with transcription and translation changes, cascade reaction increases and changes in various components of the respiratory chain, such as cytochrome oxidase, flavin dehydrogenase and cytochromes, induced when mitochondria are irradiated in isolation [32].

Therefore, we designed a tripled-blinded randomized clinical trial with 40 patients suffering from FM, based on the hypothesis that the circadian blood pressure rhythm, the perception of pain pressure and the elasticity of tissue are altered in FM sufferers. As far as we know, this is the primary research paper on the effects of PBM on circadian BP changes, pain pressure threshold and the elastic properties of tissue. FM and circadian rhythm are thought to be associated [10]; therefore, the aforementioned intervention may enhance possible treatment options for patients suffering from FM, leading to improvements in the quality of life, pain perception, quality of tissue and the socioeconomic impact of FM [13].

The research aim is to analyse changes in BP values, PPT and the elastic properties of tissue in subjects suffering from FM after a whole-body PBM treatment.

## 2. Methods

### 2.1. Study Design

This was a tripled-blinded randomized clinical trial, where subjects, therapists, evaluators and the statistician were all blinded to whether the whole-body PBM was active or in a placebo mode.

All methods used followed the Declaration of Helsinki, and ethical approval was obtained from the local Ethics Committee of the University of Granada (1044/CEIH/2020). The study has been registered on ClinicalTrials.gov (NCT05113589) and conducted in accordance with the Declaration of Helsinki. The standard protocol items of the CONSORT Statement [33,34]

### 2.2. Participants

A sample of forty-two participants who presented with FM was recruited from a private clinical practice. Participants were included in the study if they met the inclusion criteria when assessed by a research assistant, resulting in 40 participants being assessed.

Each participant had to complete a consent form before their confidential information was password-protected and stored.

### 2.3. Inclusion Criteria

i. Age: 34 to 64 years;
ii. Rheumatologist diagnosed FM based on the classification criteria of the ACR (modified 2010/2011) [35]. The following criteria must be met in order for an adult to be diagnosed with FM: (1) pain of a generalized nature in a minimum of 4 out of 5 areas; (2) a comparable degree of symptoms lasting for no less than 3 months; and (3) a score of ≥ 5 on the Symptom Severity Scale (SSS) and a score of ≥ 7 on the Widespread Pain Index (WPI); or a score of ≥ 9 on the SSS and 4 to 6 on the WPI. Additionally and importantly, the presence of other conditions or other valid diagnoses cannot be excluded in the diagnosis of FM.

### 2.4. Exclusion Criteria

The presence of any disorder of an inflammatory, neurological or orthopedic nature that may affect balance, hearing or vision. Additionally, any cognitive impairment that may impair the ability to answer questions or any muscle disorders of the fascia, such as trigger points, pain due to myofascial syndrome and neck pain.

A whole-body PBM treatment or a placebo treatment was randomly assigned to the participants who were not allowed to take part in any other FM study nor receive any other treatment while in this study. Medically prescribed medication continued and any modifications made to it were recorded; hence, the placebo effect related only to the PBM session. Any participant who had had prior treatments, namely manual therapy and physical activity, were accepted, since FM patients need continuous care.

### 2.5. PBM Therapy Program

**Figure 1:**
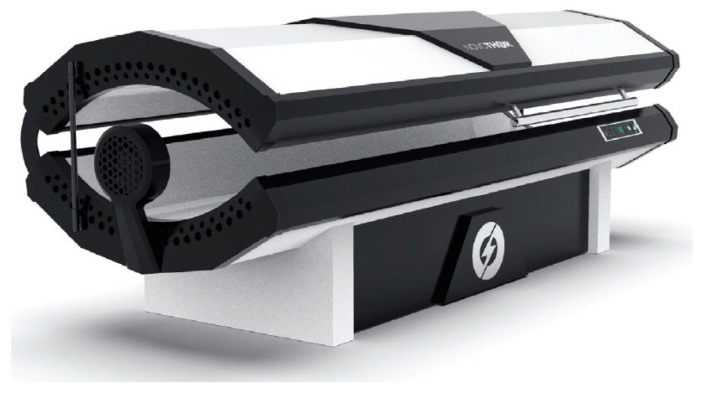
PBM bed by NovoTHOR.

A NovoTHOR^®^ whole-body light bed (Figure 1) was used to treat participants who were randomly chosen to receive treatment. The participants, either naked or in underwear, lay flat on the bed for twenty minutes three times per week for four weeks, resulting in a total of 12 treatment sessions carried out from 8 am to 16 pm. Table 1 shows the parameters of the equipment.

#### Placebo Component

**Figure 2:**
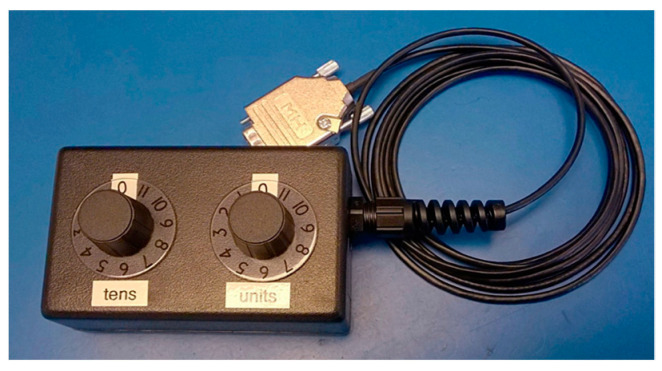
NovoTHOR randomising switch box.

The PBM bed contains a switch box (see Figure 2), which randomly assigns participants to receive an active or placebo treatment in an undetectable manner that neither the participant, the operator, nor the observers are aware of any difference in the session; hence, no other randomization is required. If the operator becomes unblinded, only the present treatment is discovered, resulting in that particular subject being excluded and not the operator. The generation of comparison groups, in a ratio of 1:1, was ensured by using a blocked randomisation system (randomly varying the block size) and for each block of ten participants, five were assigned to each leg of the trial. In the worst-case scenario, the allocation could be out of balance by as much as two.

Moreover, the participant, operator and observers wore special goggles, which were designed to accommodate glasses, that emit some red LED light inside the goggles in order to make it more difficult for them to detect whether the PBM bed was in the active or placebo mode.

Additionally, in the placebo mode, the NovoTHOR PBM bed activated heating elements, which provided the subjects with the sensation of an active treatment.

Photobiomodulation is easy to administer, safe, non-invasive, has no known side effects and has few reported contraindications [36].

### 2.6. Data Collection

**Figure 3:**
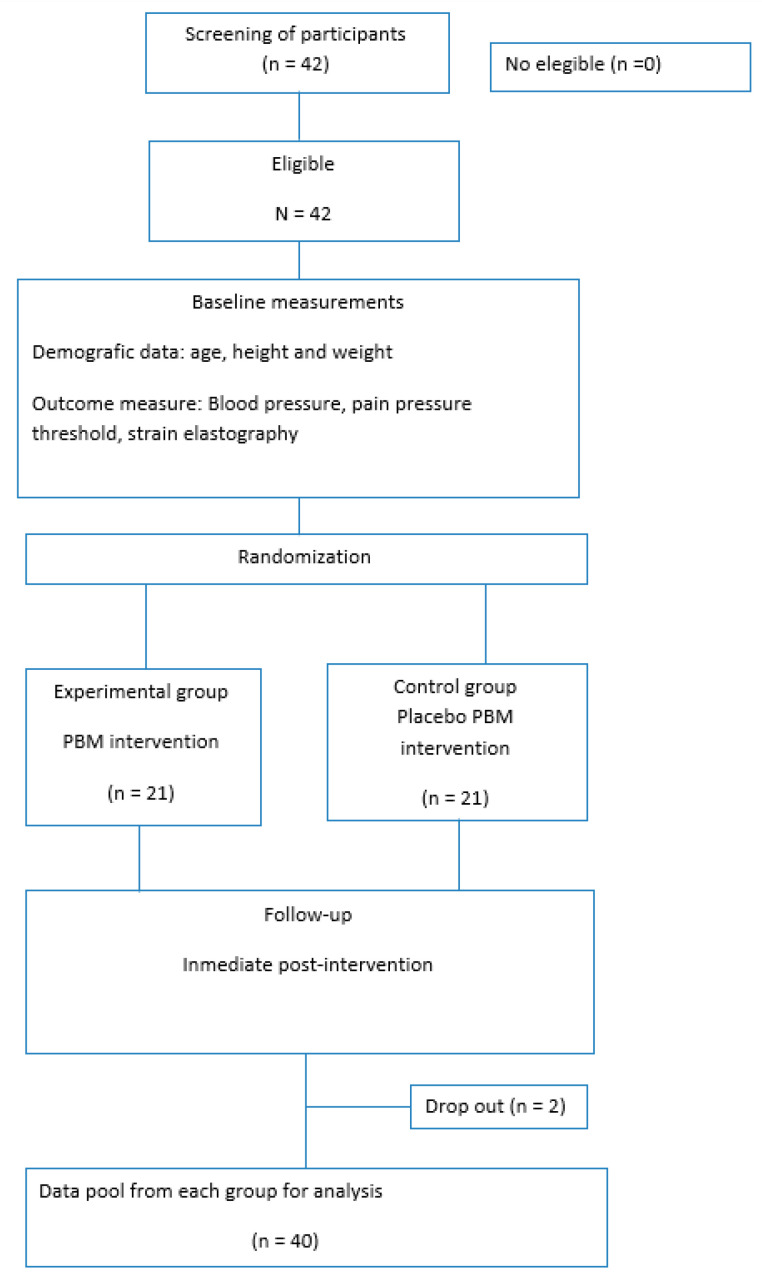
Assessment process.

Primary and secondary outcome measures were assessed at baseline (T1) and subsequently after the treatment (T2). A flow diagram illustrates these assessment times (Figure 3).

### 2.7. Primary Outcome Measures

#### Circadian BP Index

The circadian BP index (BPI) is defined as the nighttime reduction in BP relative to the mean daytime BP, and it is calculated using the following formula: 100 × (mean daytime BP−mean nighttime BP)/mean daytime BP; the mean BP is calculated as follows: diastolic BP + 1/3 (systolic BP−diastolic BP). This index was used to arbitrarily classify patients as normal dippers (ratio > 10%) or non-dippers (ratio < 10%). Recently, this allocation has been enhanced into four possible groups as follows: extreme-dippers (ratio ≥ 20%), normal dippers (ratio ≥ 10%), non-dippers (ratio < 10%) and inverse-dippers or risers (ratio < 0%, indicating a nighttime BP above the daytime mean) [23].

The guidelines published by the International Society of Hypertension (ISH) were used as the basis for measurement procedures and were taught to the patients by the research assistant [37]. Both daytime BP (at the moment of waking up between 7 am and 8 am) and nighttime BP (before going to bed between 24 pm and 1 am) were measured by the subjects over 7 consecutive days and sent to the aforementioned research assistant.

After receiving all data, the daytime and nighttime BP values for the 7 days were used to calculate the daily BP ratio and the mean diurnal/nocturnal ratio. In total, 560 BP measurements (280 daytime/280 nighttime) were carried out with the participants using validated self-measurement BP devices, following the aforementioned guidelines from the ISH and thus producing reliable values for scientific research [12,38].

### 2.8. Secondary Outcome Measure

#### 2.8.1. Pain Pressure Threshold (PPT)

A standard pressure algometer (FPK 20; Wagner Instruments, Greenwich, CT, USA), exerting up to 4 kg of pressure on 1 cm^2^ was used to assess 12 tender points in accordance with the ACR criteria. The perpendicularly positioned algometer was used to apply continually increasing pressure until pain was perceived at the following tender points: (i) occiput—suboccipital muscle insertions; (ii) low cervical—anterior aspects of C5–C7 intertransverse spaces; (iii) trapezius—midpoint of upper border; (iv) supraspinatus—origins atop the scapula spine close to the medial border; (v) paraspinous—laterally 3 cm to the midline at mid-scapula; (vi) second rib—just lateral to the upper surface second costochondral junctions; (vii) lateral pectoral—anterior axillary line at the level of the fourth rib; (viii) lateral epicondyle—2 cm distal to the epicondyles; (ix) medial epicondyle; (xi) gluteal—anterior fold of muscle at the upper outer buttock quadrants; (x) greater trochanter—just posterior to the trochanteric prominence; (xi) knees—medial fat pad adjacent to the joint line; (xii) forearm—at distal dorsal third; (xiii) thumbnail and (xiv) midfoot—dorsal third metatarsal midpoint. At these tender points, two measurements were taken, and the mean was calculated and recorded for each participant [39,40].

#### 2.8.2. SEL Measurements

A physiotherapist, with 10 years of experience and an expert in musculoskeletal imaging, used the Logiq S7 with 15 MHz linear probe (GE Healthcare, Milwaukee, WI, USA) to carry out measurements on the subjects following the orientation guidelines used for tender point recognitions [41]. Given that the longitudinal plane demonstrated both a higher intra- and inter-examiner reliability (ICC = 0.66–0.74) than the transversal one [42], the transducer was positioned longitudinally to the muscle fibers with the center of the probe over the tender and control point locations and approximately 2–5 mm of compression was applied to the tissue. The recommended compression size was evaluated using the integrated software quality control system, which visually displays one to five green bars, with the most acceptable size showing five bars. Only images with these green bars were used as per the manufacturer’s instructions. Additionally, these instructions were consulted and followed to calculate the exact raw strain value of the tissue, by using a soft, round 5mm area, with resulting levels of 0 (the softest) to 6 (the firmest); these are referred to in prior studies [43]. Intra-observer variation was minimized by calculating the mean of the three measured areas at each point.

### 2.9. Data Analysis

All analysis was carried out using SPSS^®^ Statistics version 21.0 (IBM, Chicago, IL, USA). Data distribution normality was verified using the Kolmogorov–Smirnov test. The comparison of the clinical characteristics of the PBM intervention and the placebo groups at baseline and immediately post-intervention was carried out using a two-way repeated measures MANOVA, with each moment of assessment possessing two corresponding levels and with the two intervention groups as independent factors. A *p*-values < 0.05 was considered to be statistically significant.

The Cohen d coefficient was employed to assess the size of the between group and within group effect for all qualitative variables, with greater than 0.8 being considered as large, approximately 0.5 considered as moderate and under 0.2 considered as small [44].

#### Sample Size Calculation

In reference to the outcomes of earlier randomized clinical trials [32,45] and earlier reviews [46], and in order to identify the difference between the treatment and sham groups, a standard deviation of 2.0 on the NPRS, the minimum clinically important difference [47]

## 3. Results

A total of 42 participants were recruited, but 2 participants were excluded due to not completing the proposed assessments. A final number of 40 participants were accepted and completed the baseline assessment, as shown in the flow diagram.

### Sample Characteristics

Sample characteristics are shown in Table 2. Between groups differences in PPT, SEL and BP values are shown in Table 3 and Table 4.

Circadian variations in BP showed significant differences after the PBM intervention (*p* = 0.036). When comparing PPT between groups, statistically significant differences were found in occiput (*p* = 0.039), low cervical (*p* = 0.035), trapezius (*p* = 0.037), second rib (*p* < 0.001) and medial epicondyle points (*p* = 0.006). Furthermore, there were statistically significant differences in both the trapezius and the forearm at the distal dorsal third SEL values (*p* ≤ 0.001) when comparing groups.

## 4. Discussion

This study aimed to analyse changes in the circadian variation in BP, pain pressure threshold and the elasticity of tissue, in patients with FM after a whole-body PBM treatment.

Circadian variations in BP showed significant differences after the PBM intervention (*p* = 0.036). When comparing PPT between groups, statistically significant differences were found in occiput (*p* = 0.039), low cervical (*p* = 0.035), trapezius (*p* = 0.037), second rib (*p* < 0.001) and medial epicondyle points (*p* = 0.006); nevertheless, there were no significant changes in the other points. In addition, there were statistically significant differences in both the trapezius and the forearm at the distal dorsal third. SEL values (*p* ≤ 0.001) when comparing groups, whereas no significant changes were presented in the rest of SEL assessment.

This study is the first analysing changes in circadian blood pressure values, PPT in the common tender points and strain elastography in subjects who suffer from FM; therefore, comparing these results with other studies is complicated. The presented changes may be explained given the physiological effects that PBM produces on the body’s system, including increased microcirculation, mitochondrial function, enhanced ATP synthesis and the stimulation of the mitochondrial and respiratory chain, that may influence soft tissue metabolism [27,28]. Furthermore, an increase in the activity of creatine kinase (CK), hexokinase and antioxidant production and a reduction in the release of reactive oxygen species seem to be produced after PBM [48]. Furthermore, whole-body PBM includes not only peripheral but also central stimulation, thereby including brain PBM, which is being used for a wide range of neurological and psychological conditions. An enhancement in the metabolic capacity of neurons and responses, such as anti-inflammatory, antioxidant, anti-apoptotic, neurogenesis, synaptogenesis, cerebral blood flow, oxidative stress, neuroinflammation, neural apoptosis, neurotrophic factors and neurogenesis, as well as intrinsic brain networks effects and a systemic response, has been shown after a brain PBM treatment [28], and they are also proposed within the whole-body PBM intervention. This may also explain changes in PPT and SEL measurements since it may be understood as an improvement in both the central nervous system and neuroinflammation. This produces changes in vegetative symptoms such as altering pain pressure perception and in the elasticity of tissue.

A BPI between 10% and 20% represents a “dipper”, whereas values lower than 10% represents being a “non-dipper” or an inverse-dipper (<0%). Our results showed that the BP values of the whole group are non-dippers and inverse-dippers, which is in line with a recent study which showed people suffering from chronic musculoskeletal pain to have both non-dipper and inverse dipper values [49]. Moreover, secondary hypertensive patients with endocrine abnormalities and autonomic nervous system dysfunction have also been shown to present a non-dipping pattern [23]. In this regard, Coba et al. found that when comparing pain perception and BP in healthy subjects and in patients who suffer from FM, a higher BP was associated with less pain (hypoalgesia) in patients who are healthy, but those suffering from FM show a deficiency in the descending inhibitory pain mechanism, which results in a lack of chronic pain resolution [11]. Our results showed changes in circadian BP values, as well as in PPT values, and this may be explained by the enhanced sympathetic activity caused by FM being modulated after a whole-body PBM. However, there were no significant changes in the PPT and SEL points assessed, which may be explained by an insufficient PBM treatment duration, the post-treatment assessment being carried out too soon or the lack of other factors being taken into account when assessing participants, such as sleep quality, level of daily exercise and nutritional status. Additionally, the enhanced sympathetic activity caused by FM has been proposed to induce insulin resistance and endothelial dysfunction, which have detrimental effects on cardiometabolic profiles and the progress of atherosclerosis, possibly diminishing the potential response after a PBM treatment.

### 4.1. Strengths and Weaknesses

This study, which has many strengths including being the first to report the effects of whole-body PBM on values such as BP, PPT and SEL in patients who suffer from FM, opens up new possibilities in the treatment and improvement of FM symptoms. Furthermore, a triple-blinded design was followed; thus, the results are more reliable. On the other hand, there are some limitations that must be acknowledged. The information given by the clinical measurement of blood pressure (BP) is known to be limited and its values can be influenced by other factors [12]; the small sample size used in this study means that our results have to be interpreted with caution. Finally, although the treatment was carried out during a specific range of time, the fact that there was no fixed time may be a potential limitation of the study.

### 4.2. Clinical Implications

The results obtained in our study propose whole-body PBM intervention as a new possibility in the treatment of those suffering from FM, specifically in the perception of pain measured by PPT and the elasticity of tissue measured by SEL. Changes in circadian BP were also shown after the whole-body PBM intervention, and cardiometabolic profiles, insulin resistance and endothelial dysfunction may be improved. This could be a first step in designing new whole-body PBM treatments in terms of the number of sessions with the goal of increasing the beneficial effect on the body. Furthermore, the changes produced in PPT and SEL may be consequences of an improvement in vegetative symptoms, such as neuroinflammation and central nervous system dysfunction, which are characteristic in those suffering from FM.

### 4.3. Prospective

Future research with longitudinal designs studying changes in circadian BP, PPT and SEL in tender points after treatments are needed to corroborate our findings and to analyse how these changes persist. On the other hand, cardiometabolic profiles, insulin resistance and endothelial dysfunction biomarkers, together with cortisol and melatonin levels, should be studied in order to better understand changes in circadian BP values. Furthermore, gut microbiota, metabolomics and brain neurochemistry should be further studied in subjects with FM to better understand the relationship between the axis brain-gut microbiome and circadian rhythms. Finally, more studies combining whole-body PBM treatments, exercise and nutritional strategies, such as intermittent fasting or time restricted feeding, must be carried out.

### 4.4. Conclusion

Whole-body PBM produced changes in circadian BP values, the pain pressure threshold and the elasticity of tissue after a treatment program of 12 sessions. However, more studies are needed to corroborate our findings and to better understand the underlying mechanisms.

## 5. Declarations

The trial has been registered at Clinicaltrials.gov with the following identifier: NCT05113589. Ethics approval and consent to participate: Ethical approval from the Ethics Committee of Human Research at the University of Granada, Spain (1044/CEIH/2020), was obtained for this study. All the participants accepted and signed an informed consent before beginning the study.

## Figures and Tables

**Table 1:** Specifications of NovoTHOR bed.

NovoTHOR XL Specifications	
Wavelength Red infrared LEDs Near-infrared (NIR) LEDs 50:50 ratio	660 nm 850 nm
Total number of LEDs	2880
Individual LED Power emission	0.336 W
Total Power emission	967 W
Individual LED beam area (LED lens/skin contact area)	12.0 cm^2^
Dimension of emission surface	34,544 cm^2^
Duration of Treatment	1200 s
Continuous Wave (CW) (not pulsed)	
Irradiance	0.028 W/cm^2^
Fluence	25.2 J/cm^2^

**Table 2:** Summary of sociodemographic data of the women diagnosed with Fibromyalgia.

Variable	Women Diagnosed with Fibromyalgia (*n* = 40)	
	Mean ± SD/Frequency (%)	95% CI
**Age (years)**	52.8 ± 7.90	[50.30, 55.30]
**Height (m)**	1.63 ± 0.04	[1.61, 1.64]
**Weight (kg)**	78.20 ± 18.50	[72.30, 81.10]
**BMI (kg/m^2^)**	29.40 ± 6.36	[27.30, 31.40]
**SSS**	8.55 ± 1.29	[8.13, 8.98]
**WPI**	8.13 ± 2.55	[7.31, 8.94]
**Years of diagnosed FM**	8.90 ± 2.77	[8.01, 9.79]
**Menopause status**		
**Premenopausal**	28 (70.00)	
**Postmenopausal**	12 (30.00)	

Note. Data are expressed as mean ± SD for quantitative variables and as frequency (%) for qualitative variables. Abbreviations: CI (confidence interval); BMI (body mass index); SSS (Symptom Severity Score); WPI (Widespread Pain Index).

**Table 3:** Between groups differences in PPT in tender points and BPI after the intervention (95%CI).

			Women Diagnosed with Fibromyalgia (*n* = 40)		
Variable		*Mean Values at Baseline* ± *SD*	*Mean Difference after Treatment*	95 % CI	*p*-Value SE
**Occiput**	**D**	1.51 ± 0.66	−0.273	[−1.93, 0.45]	0.039 * 0.127
	**ND**	1.50 ± 0.80	−0.111	[−2.15, 0.61]	0.066 0.134
**Low cervical**	**D**	1.37 ± 0.57	−0.088	[−1.60. 0.22]	0.749 0.114
	**ND**	1.37 ± 0.67	−0.254	[−1.51, 0.15]	0.035 * 0.134
**Trapezius**	**D**	1.94 ± 0.85	−0.101	[−1.57, 0.19]	0.409 0.121
	**ND**	1.63 ± 0.67	−0.235	[1.46, 0.11]	0.037 * 0.109
**Supraspinatus**	**D**	2.12 ± 1.04	−0.015	[−1.23, 0.06]	0.923 0.163
	**ND**	1.99 ± 0.71	−0.189	[−1.23, 0.07]	0.056 0.096
**Paraspinous**	**D**	2.37 ± 0.96	−0.200	[−1.35, 0.02]	0.144 0.134
	**ND**	2.60 ± 1.04	0.056	[−1.55, 0.18]	0.635 0.119
**Lateral pectoral**	**D**	1.36 ± 0.60	−0.034	[−1.17, 0.12]	0.116 0.114
	**ND**	1.74 ± 0.85	0.183	[−1.22, 0.07]	0.344
**Second rib**	**D**	1.25 ± 2.26	0.001	[−1.33, 0.01]	0.997 0.361
	**ND**	0.74 ± 1.01	−0.632	[−1.04, 0.22]	<0.001 * 0.109
**Lateral epicondyle**	**D**	1.59 ± 1.40	−0.072	[−1.44, 0.10]	0.697 0.185
	**ND**	1.62 ± 1.24	−0.039	[−1.47, 0.12]	0.827 0.179
**Medial epicondyle**	**D**	1.33 ± 1.00	−0.207	[−2.03, 0.53]	0.187
					0.154
	**ND**	1.41 ± 1.05	−0.505	[−1.92, 0.45]	0.006 *
					0.173
**Forearm**	**D**	1.73 ± 1.51	−0.066	[−1.85, 0.40]	0.683
					0.160
	**ND**	1.53 ± 1.14	−0.074	[−1.79, 0.36]	0.551
					0.124
**Gluteus**	**D**	2.05 ± 1.33	0.180	[−1.85, 0.40]	0.248 0.153
	**ND**	1.79 ± 1.09	0.125	[−1.35, 0.03]	0.489 0.179
**Greater trochanter**	**D**	1.81 ± 1.26	−0.153	[−2.04, 0.53]	0.340 0.158
	**ND**	1.92 ± 1.41	0.010	[−2.25, 0.68]	0.948 0.158
**Anterior Tibial**	**D**	1.49 ± 1.27	−0.123	[−2.45, 0.78]	0.467 0.168
	**ND**	1.45 ± 1.18	−0.172	[−1.67, 0.27]	0.278 0.156
**BPI**		−1.22 ± 8.26	0.025 −3.01	[−0.329, 0.380] [−0.68, 0.55]	0.886 0.036 * −0.06

Note. Abbreviations: CI (confidence interval); D (dominant); ND (no dominant); BPI (Blood Pressure Index); SE (Size Effect); SD (Standard Deviation). * Significance level: *p* < 0.05.

**Table 4:** Between groups differences in SEL in tender points after the intervention (95%CI).

			Women Diagnosed with Fibromyalgia (*n* = 40)		
Variable		*Mean Values at Baseline *± SD	*Mean Difference after Treatment*	95 % CI	*p*-Value SE
**Occiput**	**D**	2.40 ± 1.07	0.381	[0.74, 0.50]	0.136 0.12
	**ND**	2.58 ± 1.48	−0.00	[0.68, 0.55]	0.979 0.21
**Low cervical**	**D**	2.04 ± 0.87	−0.004	[0.06, 1.39]	0.808 0.73
	**ND**	2.18 ± 0.97	0.174	[0.04, 1.27]	0.469 0.62
**Trapezius**	**D**	2.35 ± 1.28	0.425	[0.0.7, 0.52]	0.087 0.09
	**ND**	2.38 ± 1.29	0.522	[1.17, 0.12]	0.028 * 0.53
**Supraspinatus**	**D**	2.23 ± 0.88	0.264	[0.46, 0.77]	0.125 0.15
	**ND**	2.04 ± 0.79	−0.146	[1.13, 0.15]	0.480 0.49
**Paraspinous**	**D**	2.87 ± 1.23	−0.935	[0.42, 0.81]	0.313 0.19
	**ND**	2.49 ± 1.16	0.337	[0.31, 0.97]	0.231 0.33
**Lateral pectoral**	**D**	1.90 ± 0.78	0.325	[0.35, 0.90]	0.082 0.28
	**ND**	2.31 ± 1.01	0.932	[1.02, 0.24]	0.079 0.39
**Second rib**	**D**	2.33 ± 0.86	0.291	[0.19, 1.08]	0.196 0.45
	**ND**	2.03 ± 0.86	−1.74	[0.16, 1.10]	0.100 0.47
**Lateral epicondyle**	**D**	1.74 ± 1.03	0.072	[1.24, 0.05]	0.697 0.60
	**ND**	1.53 ± 0.85	−0.039	[0.90, 0.35]	0.827 0.28
**Medial epicondyle**	**D**	1.57 ± 1.02	−0.165	[0.66, 0.58]	0.476 0.04
	**ND**	1.66 ± 1.00	−0.175	[0.33, 0.92]	0.572 0.29
**Forearm**	**D**	2.46 ± 1.13	0.730	[0.76, 0.47]	<0.001 * 0.14
	**ND**	2.50 ± 1.22	−0.145	[0.51, 0.72]	0.601 0.10
**Gluteus**	**D**	1.70 ± 0.68	−0.025	[0.40, 0.84]	0.877 0.21
	**ND**	1.60 ± 0.76	−0.146	[0.38, 0.86]	0.313 0.24
**Greater trochanter**	**D**	1.99 ± 1.24	0.058	[0.31, 094]	0.774 0.31
	**ND**	1.89 ± 1.12	−0.0.09	[0.68, 0.55]	0.703 0.06
**Anterior Tibial**	**D**	1.59 ± 1.08	−0.291	[0.03, 1.27]	0.342 0.62
	**ND**	1.56 ± 0.81	−0.862	[0.33, 0.92]	0.140 0.29

Note. Abbreviations: CI (confidence interval); D (dominant); ND (no dominant); SE (Size effect); SD (Standard Deviation). * Significance level: *p* < 0.05.

## Data Availability

The data sharing statement is currently not available as a secondary analysis is being performed. However, the available data can be obtained by contacting the corresponding author when the entire study is finished.
